# Immune Checkpoint Inhibitors: The Unexplored Landscape of Geriatric Oncology

**DOI:** 10.1093/oncolo/oyac119

**Published:** 2022-07-04

**Authors:** Khalil Choucair, Abdul Rafeh Naqash, Caroline A Nebhan, Ryan Nipp, Douglas B Johnson, Anwaar Saeed

**Affiliations:** University of Kansas School of Medicine-Wichita, Department of Internal Medicine, Wichita, KS, USA; The University of Oklahoma College of Medicine, Department of Internal Medicine, Division of Hematology/Oncology; Stephenson Cancer Center, Oklahoma City, OK, USA; Vanderbilt University Medical Center, Department of Medicine, Division of Hematology/Oncology, Nashville, TN, USA; The University of Oklahoma College of Medicine, Department of Internal Medicine, Division of Hematology/Oncology; Stephenson Cancer Center, Oklahoma City, Oklahoma, USA; Vanderbilt University Medical Center, Department of Medicine, Division of Hematology/Oncology, Nashville, Tennessee, USA; Kansas University Cancer Center, Department of Medicine, Division of Medical Oncology, Kansas City, KS, USA

**Keywords:** immune checkpoint inhibitors, geriatric oncology, biomarkers, immunotherapy, neoplasm

## Abstract

Cancer is classically considered a disease of aging, with over half of all new cancer diagnoses occurring in patients over the age of 65 years. Immune checkpoint inhibitors (ICIs) have revolutionized cancer treatment, yet the participation of older adults with cancer in ICI trials has been suboptimal, particularly at the extremes of age. Despite significant improvement in treatment response and an improved toxicity profile when compared with conventional cytotoxic chemotherapies, many cancers develop resistance to ICIs, and these drugs are not free of toxicities. This becomes particularly important in the setting of older adults with cancer, who are generally frailer and harbor more comorbidities than do their younger counterparts. Immunosenescence, a concept involving age-related changes in immune function, may also play a role in differential responses to ICI treatment in older patients. Data on ICI treatment response in older adult with cancers remains inconclusive, with multiple studies revealing conflicting results. The molecular mechanisms underlying response to ICIs in older cancer patients are poorly understood, and predictors of response that can delineate responders from non-responders remain to be elucidated. In this review, we explore the unique geriatric oncology population by analyzing existing retrospective datasets, and we also sought to highlight potential cellular, inflammatory, and molecular changes associated with aging as potential biomarkers for response to ICIs.

## Teaser

This review explores the unique geriatric oncology population by analyzing existing observational and trial secondary datasets to highlight cellular, inflammatory, and molecular changes associated with aging as potential biomarkers for response to immune checkpoint inhibitors.

**Implications for Practice**

Older adults with cancer represent a distinct population with myriad molecular and immune changes, as well as performance status that warrant special consideration when selecting immune checkpoint inhibitors (ICI)-based therapies. Chronological age alone does not seem to be a reliable predictor of treatment response, some studies suggest variability in ICI efficacy particularly in patients at the extremes of age. This review describes new potential approaches that integrate our understanding of the aging immune system and the age-related genetic, molecular, and metabolic changes to investigate biomarkers of response/resistance in older patients with cancer.

## Introduction

Cancer is predominantly a disease of older individuals, with estimates suggesting that over half of all newly diagnosed cancers occur in patients above the age of 65.^1,2^ However, chronological age alone, does not reliably reflect cancer treatment tolerability and prognosis.^3,4^ For immune checkpoint inhibitors (ICIs) specifically, the age-related remodeling processes of the immune system known as immunosenescence have been hypothesized to alter the efficacy and toxicity of these ICI agents in the geriatric oncology population.^5^

Older adults have historically been under-represented in trials^6,7^: data reported from the Southwest Oncology Group revealed that only a quarter of cancer clinical trials participants are 65 years or older.^8,9^ Moreover, patients over the age of 80 years represent just 4% of cancer clinical trial participants.^10^ Therefore, the current therapeutic approach for older adult patients with cancer is largely based on data derived from younger patients, despite key differences including potential decreased performance status, comorbidities, and immunosenecense.^3^

The clinical efficacy of ICIs is well established and ICIs have been approved by the US Food and Drug Administration in 19 different tumor types.^11^ Despite the significant improvements seen with ICIs, most patient experience either primary or acquired resistance to these drugs, limiting their benefit while still exposing patients to risk of rare but potentially life-threatening toxicities, termed immune-related adverse events.^3^ For older patients with cancer, this is particularly salient, as these individuals may be frailer and harbor more comorbidities than their younger adult counterparts.^12^ Thus, an urgent need exists for biomarkers of response for initial patient selection and monitoring of treatment response in older adults with cancer. In this review, we aim to explore the unique geriatric oncology population by analyzing existing retrospective datasets, and we seek to highlight potential cellular, inflammatory, and molecular changes associated with aging as potential biomarkers for response to ICIs

## Material and Methods

**Figure 1.**
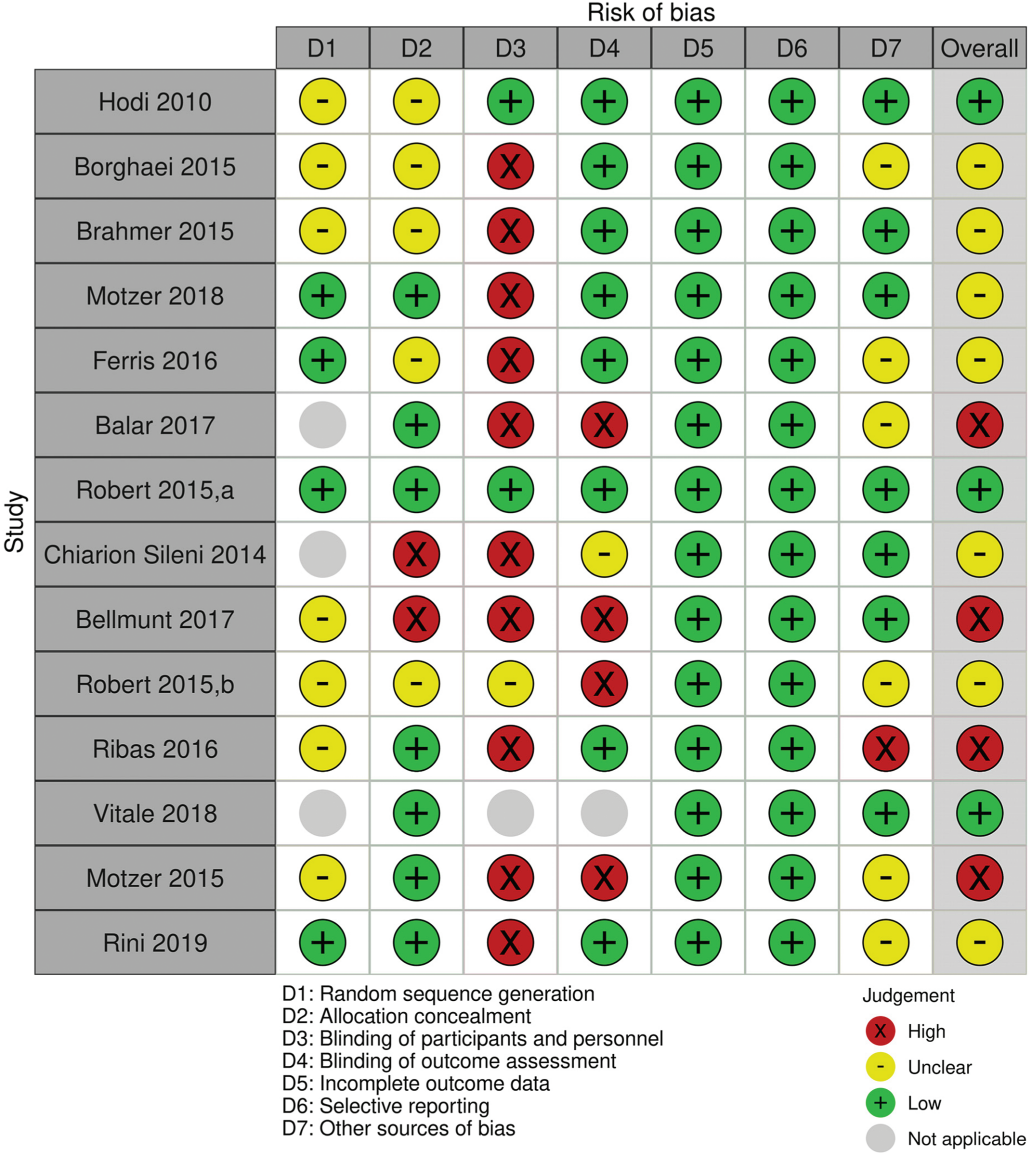
Risk of bias assessment for the randomized controlled trials included in this review: subjective assessment carried according to the Cochrane Collaboration’s tool for assessing risk of bias in randomized trials, and figure generated using the risk-of-bias visualization (robvis): an R package and Shiny web app for visualizing risk-of-bias assessments.

**Figure 2.**
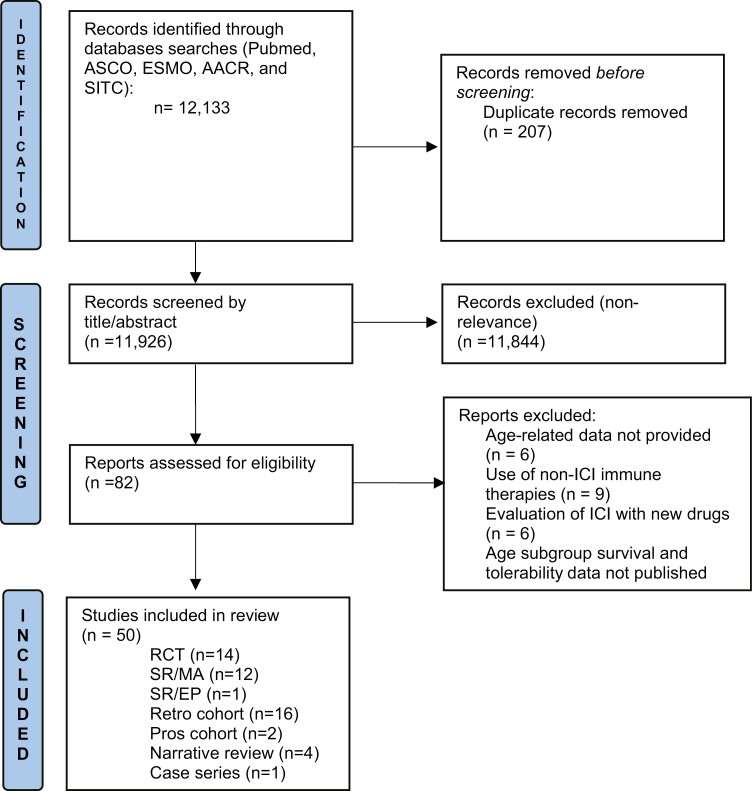
PRISMA flow diagram: selection process for the different studies included in this review. Abbreviations: ASCO, American Society of Clinical Oncology; ESMO, European Society for Medical Oncology; AACR, American Association of Cancer Research; SITC, Society for Immunotherapy of Cancer; RCT, Randomized controlled Trials; SR/MA, Systematic reviews and meta-analysis; SR/EP, systematic review and expert panel; Retro, retrospective; Pros, prospective.

We conducted a systematic review according to the PRISMA guidelines, (last updated search: December 10, 2021), to investigate ICIs in older patients with solid malignancies. The search was conducted in PubMed as well as major conference proceedings using the following query terms: (cancer OR malignancy OR carcinoma OR oncology) AND (older OR elderly OR geriatric) AND (immunotherapy OR ICI OR immune therapy OR anti-PD-1 OR anti-PD-L1 OR anti-CTLA-4). Manuscripts were included in the review if they reported the use of an ICI as a monotherapy or in combination with another ICI or chemotherapy agent in older patients with cancer (defined as ≥65 years of age). We included retrospective/prospective studies, systematic reviews, meta-analyses and clinical trial data when available. Studies were excluded if they did not meet inclusion criteria, or if they evaluated non-ICI immunotherapies (vaccines, cell-based therapies, or dendritic cells/cytokine-induced therapies). Detailed methodology including data source, selection process, risk of bias assessment (Fig. 1, Table 1) and data extraction is provided in Supplementary Material. The initial search identified a total of 12 133 studies. After applying our inclusion/exclusion criteria, 50 studies were included in the final review including 2 prospective and 16 retrospective studies, 5 reviews, 14 randomized trials, and 13 meta-analyses. Figure 2 provides the selection process flow diagram and Table 2 summarizes the studies included in this review.

**Table 1.** Summary of the clarity risk of bias tool for cohort: adapted from the CLARITY group at McMaster University and available at: http://help.magicapp.org/knowledgebase/articles/327941-tool-to-assess-risk-of-bias-in-cohort-studies.

Clarity risk of bias tool for cohort
1. Was selection of exposed and non-exposed cohorts drawn from the same population?
2. Can we be confident in the assessment of exposure?
3. Can we be confident that the outcome of interest was not present at start of study
4.Did the study either match exposed & unexposed for confounders or statistically adjust for confounders?
5.Can we be confident in the assessment of the presence or absence of prognostic factors?
6. Can we be confident in the assessment of outcome?
7. Was the follow up of cohorts adequate?
8. Were co-Interventions similar between groups?

**Table 2.** Summary of studies included in this review.

First author, year (ref)	Study design	Country	Sample size (n)	Cancer type	Intervention/arms
Randomized controlled trials (RCT)					
Hodi, 2010 (^46^)	RCT	International	676	MEL	Ipilimumab ± gp100
Borghaei, 2015 (^49^)	RCT	International	582	NSCLC	Nivolumab vs chemo
Brahmer, 2015 (^50^)	RCT	International	272	NSCLC	Nivolumab vs chemo
Motzer, 2018 (^30^)	RCT	International	1096	RCC	Nivolumab+ipilimumab vs Sunitinib
Ferris, 2016 (^51^)	RCT	International	361	HNC	Nivolumab vs chemo
Balar, 2017 (^52^)	RCT	International	374	Urothelial Ca	Pembrolizumab monotherapy
Robert, 2015 (^24^)	RCT	International	418	MEL	Nivolumab + chemo vs Nivolumab + placebo
Chiarion Sileni, 2014 (^27^)	RCT	Italy	188	MEL	Ipilimumab monotherapy
Bellmunt, 2017 (^54^)	RCT	International	542	Urothelial Ca	Pembrolizumab monotherapy
Robert, 2015 (^25^)	RCT	International	834	MEL	Pembrolizumab vs Ipilimumab
Ribas, 2016 (^26^)	RCT	International	655	MEL	ICI monotherapy
Vitale, 2018 (^33^)	RCT	Italy	389	RCC	ICI monotherapy
Motzer, 2015 (^29^)	RCT	International	821	RCC	Nivolumab vs everolimus
Rini, 2019 (^31^)	RCT	International	861	RCC	ICI+Axitinib vs sunitinib
Systematic reviews and meta-analysis (SR/MA) and expert panel (SR/EP)					
Nishijima, 2016 (^37^)	SR/MA	N/A	5265	MEL, PCa, NSCLC, RCC	ICI vs placebo ICI/Chemo vs ICI/placebo
Elias, 2018 (^38^)	SR/MA	N/A	5458	NSCLC, MEL, RCC, HNC	ICI vs chemo
Landre, 2016 (^42^)	SR/MA	N/A	687	NSCLC	Nivolumab vs chemo
Khan, 2018 (^16^)	SR/MA	N/A	3867	NSCLC	ICI vs chemotherapy
Zhang, 2019 (^17^)	SR/MA	N/A	8176	NSCLC	ICI ± chemotherapy
Zheng, 2019 (^18^)	SR/MA	N/A	4994	NSCLC	ICI vs chemotherapy
Sun, 2020 (^19^)	SR/MA	N/A	4633	NSCLC	ICI vs chemotherapy
Yan, 2020 (^20^)	SR/MA	N/A	6469	NSCLC	ICI ± chemotherapy
Ninomiya, 2020 (^39^)	SR/MA	N/A	14261	NSCLC, MEL, Gastric Ca	ICI monotherapy
Yang, 2020 (^40^)	SR/MA	N/A	23760	MEL, GU, SCLC, Gastric Ca, NSCLC, HNC,	ICI monotherapy vs non-ICI therapy
Kasherman, 2020 (^41^)	SR/MA	N/A	13314	NSCLC, MEL, HNC, GEJ, RCC, Prostate Ca, SCLC and Bladder Ca	ICI monotherapy
Landre, 2020 (^43^)	SR/MA	N/A	9647	NSCLC, MEL, HNC, GEJ, RCC, Prostate Ca, SCLC and Bladder Ca	ICI monotherapy
Gridelli, 2005 (^8^)	SR/EP	International	N/A	NSCLC	N/A
Retrospective (Retro) and prospective (Pros) cohort studies					
Elkrief, 2020 (^48^)	Retro. cohort	France/Canada	381	NSCLC	ICI monotherapy
Herin, 2018 (^47^)	Retro. cohort	France	220	Diverse solid tumors	ICI monotherapy
Gomes, 2021 (^4^)	Pros. cohort	UK	140	NSCLC, MEL	ICI monotherapy
Betof, 2017 (^23^)	Retro. cohort	USA	254	MEL	ICI monotherapy
Rai, 2016 (^53^)	Retro. cohort	USA/Australia	283	MEL	ICI monotherapy
Kugel, 2018 (^56^)	Retro. cohort	USA	538	MEL	ICI monotherapy
Ibrahim,2018 (^22^)	Retro. cohort	France	99	MEL	ICI monotherapy
Nebhan, 2021 (^36^)	Retro. cohort	International	928	NSCLC, MEL, GU	ICI monotherapy
Youn, 2020 (^13^)	Retro. cohort	USA	1256	NSCLC	ICI monotherapy
Lichtenstein, 2019 (^15^)	Retro. cohort	USA	245	NSCLC	ICI monotherapy
Perier-Muzet, 2018 (^21^)	Retro. cohort	France	92	MEL	ICI monotherapy
Weber, 2017 (^28^)	Retro. cohort	International	576	MEL	ICI monotherapy
Corbaux, 2019 (^34^)	Retro. cohort	France	410	NSCLC, MEL, GU	ICI monotherapy
Sattar, 2019 (^35^)	Retro. cohort	Canada	78	NSCLC, MEL, RCC	ICI monotherapy
Erbe, 2021 (^79^)	Retro. cohort	USA	64859	Breast, CRC, HNC, Bladder, ECa, MEL, NSCLC, RCC,	N/A
Moreira,2018 (^80^)	Retro. cohort	Germany	10	MEL	ICI monotherapy
Ferrara, 2021 (^81^)	Retro. cohort	France	83	NSCLC	ICI vs chemo
DeGiorgi,2019 (^32^)	Pros. Cohort	International	313	RCC	ICI monotherapy
Narrative reviews and case series					
Gomes 2018 (^7^)	Review	UK	N/A	NSCLC	N/A
Daste, 2017 (^44^)	Review	N/A	N/A	N/A	N/A
Granier, 2021 (^3^)	Review	France	N/A	N/A	N/A
Ferrara, 2017 (^45^)	Review	France	N/A	NSCLC	N/A
Johnpulle, 2016 (^55^)	Case series	USA	3	MEL	ICI monotherapy

Abbreviations: RCT, randomized controlled trial; MEL, melanoma; NSCLC, non-small cell lung cancer; Chemo, chemotherapy; RCC, renal cell cancer; HNC, head and neck cancer; Ca, cancer; ICI, immune checkpoint inhibitor; SR, systematic review; MA, meta-analysis; EP, expert panel; N/A, not applicable; GU, genitourinary; SCLC, small cell lung cancer; GEJ, gastro-esophageal cancer; Retro, retrospective; Pros, prospective; USA, United States of America; UK, United Kingdom; CRC, Colorectal Cancer.

## Immune Checkpoint Inhibitors in Older Adult Patients (≥65 Years)

To date, data regarding ICIs in older patients with cancer come from observational studies and subgroup analyses of phase III pivotal clinical trials. In the following sections, we review the published efficacy and toxicity data of ICIs in older patients with cancer, with a focus on tumor types most commonly utilizing ICI in standard treatment regimens: non–small cell lung carcinoma, melanoma, and renal cell carcinoma.

## Non-Small Cell Lung Carcinoma

Real-world data from the Surveillance, Epidemiology, and End Results-Medicare linked database was used to identify 1256 patients aged ≥65 years with NSCLC treated with nivolumab or pembrolizumab in the adjuvant or metastatic setting, in first and later lines of treatment.^13^ The study revealed that the number of comorbid conditions, rather than patient age, was significantly associated with an increased hazard of death (HR = 1.40; 95% CI: 1.15-1.70), with no statistically significant impact for the line of therapy. Notably, no differences were seen in survival and prognosis between different age groups.^13^ In terms of toxicity, a prospective study by Gomes et al evaluated ICI-related toxicity in older patients treated with ICI monotherapy (ELDERS study).^4^ Patients ≥70 years of age with NSCLC and melanoma were compared with younger patients in terms of frailty and incidence of immune-related adverse events (irAEs). Results from this study revealed that while the older cohort had significantly higher comorbidity burden, no significant differences were seen in the incidence of high-grade irAEs (grades 3-5)^14^ between older and younger patients (*P* = .353). While these studies showed no effect for older age on response to ICI or on ICI-associated toxicities, another retrospective study evaluated 245 patients with NSCLC-treated PD1/PD-L1 inhibitors, revealing that both median progression-free survival (mPFS) and overall survival (mOS) differed by age (mPFS for <69 years: 2.53 months vs 3.75 months for ≥70 years, *P* = .055; and mOS for <69 years: 14.56 months vs. 12.92 months for ≥70 years, *P* = .011, respectively), while rates of toxicity remained similar across age groups.^15^

In a meta-analysis of randomized controlled trials comparing anti-PD-1/PD-L1 monotherapy and chemotherapy in the treatment of advanced NSCLC, Khan et al selected 7 trials and revealed a better OS, PFS, and objective response rate (ORR) for ICI (pooled HRs of 0.72 (*P* < .00001), 0.84 (*P* < .02) and odds ratio 1.52 (*P* < .02), respectively). Subgroup analyses showed improved OS for ICI in patients above age 65 years (*P* = .006), but not for patients above 75 years (*P* = .56). For PFS, there was no significant association with age, in patients over 65 (*P* = .14) or 75 years (*P* = .45).^16^ Similarly, another meta-analysis compared the efficacy of ICIs between older and younger patients with advanced NSCLC^17^: in 12 eligible trials involving 8176 patients, the overall HR for patients <65 years was 0.75 (95% CI: 0.65-0.87) compared with 0.81 (CI 0.72-0.92) for older patients, highlighting that ICIs can improve OS for older patients with advanced NSCLC. Given significant data heterogeneity in patients ≥65 years, a subgroup analysis using age 75 as a cut-off was conducted and revealed that patients >75 years had no superior benefit from ICIs (*P* = .520). In a more recent meta-analysis of data from 8 trials, the efficacy and safety of ICIs in 4994 patients with NSCLC was compared across different age groups: a significant OS benefit was reported with ICI treatment compared with chemotherapy among both younger (<65years, HR 0.73; 95% CI: 0.61-0.89) and older adult patients (≥65, HR 0.74; 95% CI: 0.59-0.93).^18^ However, there was no statistically significant prolongation of OS among patients with NSCLC older than 75 years compared with chemotherapy, consistent with findings from prior studies. The study also revealed fewer adverse events of ICIs compared with chemotherapy, irrespective of age.

Consistent with previous findings, 2 other meta-analyses revealed comparable efficacy and tolerability of ICIs between patients with NSCLC younger and older than 65 years of age: Sun et al analyzed data from 8 phase II/III studies that included 2662 patients <65 years and 1971 patients ≥65.^19^ The efficacy of anti-PD-1/PD-L1 agents was comparable between the 2 groups for OS but not for PFS. Finally, Yan et al evaluated the impact of age on ICI efficacy when combined with other therapies, in a meta-analysis of 10 trials involving 5487 patients with NSCLC. The study revealed a statistically comparable OS and PFS advantage for ICI combinations in younger and older patients.^20^

In summary, several studies have reported tolerability and efficacy of ICIs in patients with NSCLC ≥65, but efficacy remains uncertain in patients >75. Collectively, these observations highlight that additional work is needed to fully understand the mechanism of immune response in aging to help derive predictive biomarkers for older adults with cancer.

## Melanoma

In a single-center retrospective study, the clinical outcome of older patients treated with ipilimumab, nivolumab, or pembrolizumab was evaluated: patients older than 65 years had longer median OS (not reached vs 10.1 months; *P* = .009) and PFS (4.8 vs 3.4 months; *P* = .04) compared with younger patients.^21^ This age impact was more pronounced for patients treated with anti-PD-1 agents compared with anti-CTLA-4. The efficacy of ICIs was also evaluated in another retrospective study involving 99 patients with metastatic melanoma >75 years, treated with pembrolizumab, nivolumab, or ipilimumab.^22^ The median OS was not reached for pembrolizumab versus 8.7 and 23 months for ipilimumab and sequential ICIs, respectively. In a larger retrospective study by Betof et al, the efficacy of anti-PD-1/anti-PD-L1 agents was investigated across different age subgroups in 254 patients with melanoma, including 65 patients aged 65-74 and 47 ≥75.^23^ Median OS was similar between the different subgroups.

In the phase III CheckMate-066 study evaluating nivolumab vs dacarbazine in untreated *BRAF* wild-type melanoma, 52% of patients were over 65 years, and 16% were aged over 75.^24^ Clinical benefit of nivolumab was seen across different age groups, especially in patients >75 (HR 0.25). In the phase III KEYNOTE-006 study evaluating pembrolizumab versus ipilimumab in advanced melanoma, 29% of patients were >65 years.^25^ Across the different age groups, the risk of death was similar between the 2 arms. In a pooled analysis of patients with melanoma from various early-phase trials evaluating pembrolizumab, ORR was not statistically different between patients <65 years and ≥65 years.^26^ Finally, data from the Italian Expanded Access Program (IEAP), which evaluated ipilimumab in 193 patients >70 years, revealed a comparable median OS (8.9 and 7.0 months; *P* = .17) and median PFS (4.0 and 3.7 months; *P* = .33) between patients >70 and ≤70 years, respectively.^27^

Regarding toxicity, pooled analysis of phases I-III clinical trials evaluating ipilimumab or nivolumab revealed comparable incidences of irAE in patients ≥70 years compared with patients <70 years.^27,28^ Sileni et al, found that among patients >70 years treated with ipilimumab, 36% of all AE were irAEs, compared with 33% in patients ≤70 years. Similar findings were reported in patients with advanced melanoma treated with nivolumab.^28^

In summary, available data evaluating ICI therapy in patients with melanoma reflects similar outcomes in older and younger patients with regards to efficacy and tolerability.

## Renal Cell Carcinoma

In phase III CheckMate-025 study evaluating nivolumab vs everolimus in previously treated patients with metastatic renal cell carcinoma (RCC), the risk of death was significantly reduced in favor of nivolumab in patients aged 65-74 years (HR 0.64; 95% CI 0.45-0.91) but not in patients ≥75 years.^29^ In the phase III CheckMate-214 study comparing the ipilimumab/nivolumab combination to sunitinib in untreated patients with metastatic RCC, the HR of death was 0.86 for the ICI combination (95% CI: 0.53-0.82) in patients aged 65-74 years, and 0.97 (95% CI 0.48-1.95) in patients ≥75 years.^30^ In the phase III KEYNOYE-426 trial comparing pembrolizumab plus axitinib to sunitinib in the first-line treatment of patient with advanced RCC, there was a significant reduction in the risk of death (HR 0.59; 95% CI: 0.36-0.97) for patients ≥65 years.^31^ In a subgroup analysis of older adult patients with metastatic RCC treated with nivolumab from the IEAP, ORR were similar in both the overall population and the subgroup of patients ≥75 years.^32,33^ Similarly, patients ≥70 years and those ≥75 years had a similar OS at 6, 12, and 18 months compared with younger patients. Data from the IEAP report for nivolumab revealed a tolerability profile in older patients that was consistent with that of the general population^33^: while treatment-related AEs were reported in 33% of the general population, rates were 37% in patients ≥70 years of age, and 40% in patients ≥75 years.

In summary, data regarding ICI in patients with RCC aged 65-75 years consistently reveals favorable efficacy and tolerable toxicity profiles comparable to that of a younger or general oncology population, but remains equivocal for patients ≥75 years, highlighting the need for further focused studies in older adults.

## Pooled Data Across Different Cancer Types

In a retrospective study of 410 adult patients with different tumor types (lung, melanoma, and genitourinary) treated with single-agent ICI, age did not significantly correlate with OS or PFS outcomes.^34^ Similarly, grades 3-4 irAEs were not statistically different between older (≥65 years) and younger patients (*P* = .87). In a similar retrospective analysis of patients with advanced solid tumors (melanoma, NSCLC, and RCC) treated with single-agent ICI in Canada, patients <65 years, 65-74 years and ≥75 years had similar ORRs (*P* = .58*5*).^35^ Survival analysis further demonstrated a median OS of 28 months for patient <65 years versus 17 months for patients aged 64-74, with the estimated survival probability not reaching 50% in the age ≥75 years. There were no statistically significant differences in terms of irAEs across the different age groups.^35^ Lastly, a multicenter international cohort study reported clinical outcomes and toxicities of single-agent ICIs among patients aged ≥80 years with cancer.^36^ The study included 928 patients treated across 18 academic centers in the US and Europe. The most common tumor types represented included NSCLC, melanoma and genitourinary tumors. Within histology-specific cohorts, clinical outcomes were similar across age subgroups (aged <85 versus ≥85 years). There was no significant difference in the rate of irAEs among patients aged <85, 85 to 89, and 90+ years. Overall, real-world data from observational studies in older adult patients with cancer treated with single-agent ICIs strongly suggest that treatment with ICI may be effective and well-tolerated among this patients population.^34-36^

Several meta-analyses have also reported on the efficacy and tolerability of ICIs in older patients with cancer.

*Patients 65-70 years old*: In an early meta-analysis of data from nine randomized controlled trials (5265 patients) evaluating ICIs in patients with diverse malignancies, ICIs improved OS and PFS comparatively in both younger and older groups, and across different tumor types.^37^ Similar findings were reported in a meta-analysis of 9 phase II/III clinical trials (5458 patients) that specifically evaluated PD-1/L1 inhibitors in patients with metastatic solid tumors <65 vs ≥65 years of age.^38^ The study revealed comparable HR for death and progression across both age groups. In another meta-analysis, 24 randomized trials including 8157 patients ≤65 years and 6104 patients >65 years with multiple solid tumors treated with ICIs were evaluated.^39^ Analysis revealed non-statistically different pooled HR of death between younger and older patients. In a study to evaluate the difference in survival benefit of ICIs between sex, age (<65 vs ≥65 years), or performance status (Eastern Cooperative Oncology Group [ECOG] 0 and ≥1), 37 phase II/III (23760 patients) were analyzed.^40^ The pooled OS HR demonstrated that ICIs-induced survival benefit independent of sex, age, or ECOG. This was consistent across subgroup analyses by cancer type, line of therapy, and ICI agent.

*Patients ≥75 years old:* In a meta-analysis of 19 trials involving ICIs (monotherapy or in combination with other agents), the benefit of ICIs was evaluated in 13 314 patients (*n* = 6064, age ≥65 years; *n* = 7250, age <65 years) with advanced cancer.^41^ The analysis revealed no significant treatment-age interaction (*P* = .27), with similar findings when stratifying at age cut-offs of 75 years (*P* = .72). In another meta-analysis of 15 phase III clinical trials using ICIs (monotherapy or combination) vs standard therapy in patients (*n* = 9647) with advanced solid tumors, OS was compared between older (≥75 years; *n* = 906) and younger (<75 years; *n* = 8741) patients.^42,43^

Overall, studies have revealed that patients aged 65-75 years respond as well as subjects <65 years.^37,38,44-48^ However, in patients >75 years, data remains inconclusive and potentially histology-specific: higher rates of primary resistance to ICIs in older patients with cancer has been observed in phase III pivotal clinical trials involving patients with lung cancer, metastatic clear cell renal cancer and cancer of the upper aero-digestive tract.^30,42,49-51^ On the contrary, in patients with metastatic melanoma or advanced bladder cancer, no age difference for response to these treatments has been observed: in both cancer types, clinical responses in subjects over 70 or 75 years of age have been observed in a comparable manner to younger subjects.^23,24,27,52-54^ Notably, clinical responses have been reported in metastatic melanoma after treatment with ICIs among patients over 90 years of age.^22,55,56^ While fewer studies have used a cut-off of 75 years have been done, and are thus inconclusive,^30,42,49-51^ an evaluation of a large, multicenter cohort of cancer patients over the age of 80 suggests that ICIs have a good efficacy and tolerable toxicity profile in older adults.^36^

The potential difference in ICI treatment response among patients over 75 years old is ripe for biomarker development to prioritize effective treatment and to spare potential non-responders unnecessary therapy

## Comprehensive Geriatric Assessment Tools for Treatment Personalization

In light of the conflicting evidence regarding ICIs efficacy in older patients with cancer, namely at extremes of age (≥75 years), the need for treatment individualization becomes more pressing. Clinically this can be achieved by evaluating older patients for frailty using a comprehensive geriatric assessment (CGA) for patients’ selection, as recommended by the International Society of Geriatric Oncology (SIOG).^57^ In fact, the SIOG has deemed the ECOG and Karnofsky performance status to be non-specific as they generally overestimate functioning status of older patients and may not be able to predict treatment toxicity in this patient population.^57^ In studies involving chemotherapy for example geriatric assessment has been shown to predict severe treatment-related toxicity, has been associated with survival outcomes, and ultimately affected treatment choice and intensity.^58-60^

For ICIs, only one of the studies in this review assessed for frailty in relationship development of immune-related adverse events, using the Geriatric-8 (G8) screening tool (The ELDERS study; *n* = 140^4^), and none of the other studies provided data on CGA, measure of frailty and toxicity in older patients with cancer. This is of utmost relevance given prior reports of frailty being associated with specific T-cell subset profiles, suggesting that immunosenescence may be more linked to functional age rather than chronologic age.^61^

The G-8 screening tool can help identify frail older patients with cancer requiring geriatric assessment and tailoring of cancer treatment, while also preventing under-treatment of fit older patients.^62,63^ While multiple CGA tools exist, the SIOG expert panel does not recommend one tool over another.^57,64^

## Limitations

The studies included in this review carry several limitations: first, and as delineated earlier there are no randomized clinical trials dedicated to evaluating ICIs in older patients with cancer, thus limiting our ability to clearly examine the outcomes associated with ICI use in this rather unique population with exclusive aging-related conditions. Most of the trials data presented in this review were derived from subgroup analyses of pivotal trials that evaluated ICIs. The remaining sources of information consisted of observational cohort studies and systematic reviews, thus limiting the ability of oncologists to derive direct markers of response and/or clear association between age and ICI treatment outcome. Similarly, in terms of individualization of therapy, CGA is the cornerstone of geriatric oncology, and yet was reported in only one of the studies, highlighting the very limited use of this tool in ICI studies. Taken together, these gaps in the literature pave the way for future research opportunities and highlight the need for reliable markers of response to ICIs in older patients with cancer: the identification of such markers, as highlighted in the next section, could allow the effective design and implementation of prospective, marker-based trials targeting the geriatric cancer patients population.

## Characteristics of the Older Adult Population: Finding the Achilles’ Heal for ICI Efficacy

In order to define potential drivers of response to ICIs in older patients with cancer, it is important to consider the unique characteristics of this population.

1) *The aged immune system: “inflammaging” and immunosenescence*: Immunosenescence refers to the effects of aging on the immune system.^6^ It has been shown to result in immune dysregulation within both cellular and humoral immunity, with depletion of lymphocyte reserves, fewer CD4+ and CD8+ T cells, decreased diversity of regulatory and memory T cells, and an overall increased pro-inflammatory state.^37,65,66^ It has been well documented that pro-inflammatory states result in decreased response to ICI: increased inflammation in the tumor microenvironment (TME) results in upregulation of several immune checkpoint molecules, while simultaneously increasing conversion to regulatory T cells that secrete immunosuppressive molecules (TGF-β, IFNγ, and IL-10), ultimately resulting in immune escape and cellular immune anergy.^67,68^ Clinically, older adults with melanoma treated with ICIs have demonstrated reduced levels of tumor-infiltrating lymphocytes, which in turn could confer worse survival.^56,69,70^ Newer data support a correlation between frailty of older adults and specific T-cell subset profiles, suggesting that immunosenescence may be more linked to functional age rather than chronologic age.^61^ Immunosenescence is then a potentially key, albeit poorly exploited, phenomenon in determining response to ICIs in older patients with cancer.

A defining feature of immunosenescence is that of the chronic inflammatory state, also referred to as “inflammaging,” which has been linked to cancer and other diseases.^71,72^ Biochemically, this is reflected by higher serum levels of IL-6, CRP, and TNF-α, even in healthy older adults (≥65 years).^73^ Inflammation-associated genomic instability is a documented precursor of cancer development.^74^ Several pro-inflammatory pathways are well-described in carcinogenesis, including NF-κB, IL-6/STAT3, COX-2/PGE_2_, IL-23/Th17, and AP-1 pathways.^75-77^ Targeting these pathways has been suggested as a potential strategy to prevent and treat cancer.^78^ A recently published manuscript examined the correlation between age and predictive markers of ICIs response, observing increased TMB and decreased T-cell receptor diversity with aging^79^
In the setting of ICI therapy, studies have attempted to elucidate potential inflammatory biomarkers of response. In one study, loss of surface markers CD27 and CD28 or expression of Tim-3 and CD57 on peripheral T cells was associated with resistance to ICIs.^75^ Similarly, increased percentages of circulating CD8+ T cells expressing markers of senescence (CD28^-^/CD57^+^/KLRG1^+^) has been associated with resistance to ICI therapy and poor survival in patients with advanced NSCLC.^45,80^ Thus, some correlative evidence suggests that increased levels of specific circulating inflammatory and senescence markers may portend poorer response and/or resistance to ICIs.

2) *Defective DNA repair and increased immunogenicity with aging*: the aging process has been closely associated with changes in genes of the DNA damage response (DDR) pathway that appear to underlie both aging and cancer development.^81-83^ Evidence suggests that mutations in precancerous lesions not only display a high proliferation rate but also exhibit persistent DNA damage, known as replication stress.^84,85^ Both carcinogenesis and age-associated replication stress have a number of common features, including increased numbers of stalled and collapsed replication forks, deregulated replication origins, and elongating replication forks, all of which contribute to the accumulation of DNA damage. Accumulated DNA damage translates into the formation of neo-antigens that are meant to be recognized by the host immune system, and eliminated.^86^ While deficiency in DDR illustrates the complex interaction between cancer, aging and potential sensitivity to ICIs, half of DDR-deficient tumors are refractory to ICIs, and it remains unclear which mutations may promote immunogenicitym, in which cancer types, and under which host factors.^86^

Another example is the aging-related reduced binding of replicative helicase mini-chromosome maintenance complex 2-7 (MCM2-7) at replication origins, which induces replication stress and genome instability.^87^ Failure of the DNA helicase-mediated replication fork resumption is known to be associated with cancer predisposition.^84,88^ Well characterized in oncology, the BRCA protein represents an example linking homologous recombination (HR) repair proteins, replication stress and cancer.^89^ The mutation of *BRCA1* in mammary epithelial cells results in tumor formation.^90^*TP53* is another example of the common, yet poorly understood mechanisms underlying aging and cancer. The p53 tumor suppressor acts as an upstream regulator of the DDR pathway, while also being the most commonly mutated gene across different tumor types.^82^ Collectively, these data support a potential hypothesis that the differential response to ICIs in older adults with cancer could also be related the aging-related transcriptomal signatures underlying the DDR pathway that remain to be elucidated.

3) *Metabolic changes with aging:* Aging-related metabolic changes may also play a role in carcinogenesis. Both glutamine and glucose metabolism commonly underlie carcinogenesis and aging: increased glucose metabolism has been tightly linked to aging, and targeting the pathway (via caloric restriction/fasting) has been shown to slow the process of aging in mouse models.^91-94^ Similarly, increased glutamine pathway activation and its resulting by-product citrulline and α-ketoglutarate (both feed into the glucose metabolism pathway), has also been linked to aging and carcinogenesis.^95-97^ The oxidative stress pathway can also change with aging. Age-related production of reactive oxygen species and hydrogen peroxide exhibit a mutagenic potential that induces DNA damage, subsequent mutations, and the expression of potential neo-antigens.^98,99^ Furthermore, it has been hypothesized that hydrogen peroxide-induced DNA damage exhibits a “field effect” leading to changes in the composition of the TME that contribute to local inflammation, tumor escape of the immune system, and cancer metastasis.^100-103^

Taken together, there is substantial basic research to suggest that malignancy in older adult patients may exhibit features that distinguish them from younger patients, including inflammation, defects in the DDR pathway, and metabolic changes and carcinogenesis. An understanding of these processes may help generate potential biomarkers of response to ICIs in this population.

## Potential Biomarkers of Response to ICIs in Older Adults: the Unexplored Landscape

Many potential unexplored avenues for biomarkers emerge in this patient population. To advances in large-scale genomic/transcriptomic technologies, it is conceivable that exploring potential signatures using large databases in older patients and comparing those to younger patients may yield molecular signatures of predictive interest. For example, differential expression frequency of DDR genes, between younger and older patients with cancer may reveal predictive signatures of interest. Additionally, differences in the TME of older vs younger patients may be evaluated using RNA-sequence datasets to explore differences in cell type composition of TMEs. This could also be expanded to include the differential expression of immune checkpoint-related genes in both older and younger patients.

Building on the available evidence of age-driven metabolic changes in older vs younger patients and the established relationship between metabolic changes and tumorigenesis, differences in gene expression for specific metabolic pathways also warrant investigation.

Although further work is necessary to understand how processes like inflammaging and immunosenescence translate into clinically relevant circulating biomarkers, the existing preclinical data suggests reason for optimism. Such biomarkers have potential to provide reproducible and minimally invasive markers of treatment prediction in a particularly large, growing, and understudied patient population.

## Conclusion

Immune checkpoint inhibitors continue to improve outcomes for patients with cancer. As individuals’ life expectancy increases, the geriatric oncology population will continue to grow, a population with myriad molecular and immune changes as well as performance status and comorbidities, which warrant special consideration when selecting treatment. Patient-clinician discussions about treatment, including ICI-based therapies, are thus particularly important for older patients to optimize therapeutic options that maximize response while minimizing toxicity, especially at the extremes of age (>75-80 years old). A new approach, based on integrating our understanding of the aging immune system and the age-related genetic, molecular, and metabolic changes is critically needed. In this review, we have described such pathways that could constitute working paths for investigating biomarkers of response in older patients with cancer.

## Supplementary Material

oyac119_suppl_Supplementary_Material

## Data Availability

No new data was generated in the development of this review article; thus data sharing is not applicable.
