# Supplementary material for: Immune Checkpoint Inhibitors: The Unexplored Landscape of Geriatric Oncology
Source: Oncologist. 2022 Jul 4;27(9):778–89. doi: 10.1093/oncolo/oyac119 (PMC9438919; doi:10.1093/oncolo/oyac119)
Supplement: oyac119_suppl_Supplementary_Material [file oyac119_suppl_supplementary_material.doc]

**Supplementary methods**

*Data Source:* We searched PubMed database as well as the archives and proceedings from the American Society of Clinical Oncology (ASCO), the European Society of Medical Oncology (ESMO), the American Association for Cancer Research and the Society for Immunotherapy of Cancer (SITC), for studies involving ICIs in older patients with solid malignancies, from the inception of each database to December 10, 2021. For trials of relevance, we only included the most complete and recent trial when duplicate publications were identified. We included cohort studies (retrospective and prospective), narrative reviews, systematic reviews, meta-analyses and clinical trial data when available. Two investigators (K.C. and A.N.) independently retrieved all the related studies in the databases and excluded duplicate publications. The search query terms were used as follows: (cancer OR malignancy OR carcinoma OR oncology) AND (older OR elderly OR geriatric) AND (immunotherapy OR ICI OR immune therapy OR anti-PD-1 OR anti-PD-L1 OR anti-CTLA-4). We also reviewed abstracts and presentations from major conference proceedings as described above up to December 10, 2021 to ensure that no additional studies were overlooked.

*Selection Criteria*: Manuscripts were included in the review if: i) they reported the use of an ICI, whether as a monotherapy or in combination with another ICI agent or non-immune systemic therapy (chemotherapy, small molecules, radiation, etc...) as long as ICI was the investigated intervention (*vs.*ICI as standard of care with a new drug being the investigated intervention); ii) ICIs includes anti-PD1, anti-PD-L1, CTLA-4 or their combination; iii) involved older patients with cancer (defined as ≥65 years of age); iv) provided clear age-related data and subgroups when applicable iv) provided age-related survival and tolerability/toxicity data based on clear age cut-off. Two independent investigators (K.C. and A.N.) screened each reference by their titles and abstracts first, to elect potentially relevant articles meeting the pre-defined inclusion criteria, and then looked through the full text of relevant articles from the first selection. All disagreements about selection between the two investigators were discussed and resolved by all investigators. The PRISMA flow diagram summarizing the selection process is described in *Supplementary Figure S1*.

*Risk of Methodological Bias Assessment*: For RCTs, two independent investigators (K.C. and A.N.) subjectively evaluated the quality of all studies according to the Cochrane evaluation handbook of RCTs (5.1.0), which includes random sequence generation, allocation concealment, blinding of participants, personnel and outcome assessment, incomplete outcome data, selective reporting, other bias, and overall assessment, then categorized it into three levels of “Low” for a low risk of bias, “High” for a high risk of bias and “Unclear”. “Not applicable” was also used when the assessment criteria did not apply for a specific study (*Supplementary Figure S2*). For cohort studies the CLARITY group risk of bias tool for cohorts was used to assess for risk of bias (<http://help.magicapp.org/knowledgebase/articles/327941-tool-to-assess-risk-of-bias-in-cohort-studies>). A summary of the tool items is provided in *Supplementary table S2*.

*Data Extraction*: Three investigators (K.C., A.N., and A.S.) independently performed data extraction and recording in a standard form. The following basic information was acquired from each included study: i) Study characteristics: first author, publication time, and study design; ii) Study population: Sample size of each study and cancer type(s), and iii) when applicable description of intervention/study arms. For age subgroups-related data from the pivotal ICIs trials, we investigated the available supplementary material to obtain the relevant information when provided.
